# The timing of death in patients with tuberculosis who die during anti-tuberculosis treatment in Andhra Pradesh, South India

**DOI:** 10.1186/1471-2458-11-921

**Published:** 2011-12-13

**Authors:** Subbanna Jonnalagada, Anthony D Harries, Rony Zachariah, Srinath Satyanarayana, Shailaja Tetali, G Keshav Chander, Srinivas Rao, Ranganadha Rao, Sundaresh Peri, Raghupathy Anchala, Nanda K Kannuri

**Affiliations:** 1 LEPRA India - Blue Peter Public Health & Research Centre, Near TEC Building, Cherlapally, Hyderabad 501301, Andhra Pradesh, India; 2 International Union Against Tuberculosis and Lung Disease (The Union), 68 boulevard Saint Michel, Paris 75006, France; 3 Department of Clinical Research, London School of Hygiene and Tropical Medicine, 50 Bedford Square, London WC1B 3DP, UK; 4 Medecins Sans Frontieres, Medical Department (Operational Research), Operational Center, Brussels, Luxembourg; 5 International Union against Tuberculosis and Lung Diseases (The Union), South East Asia, Regional Office, C-6, Qutub Institutional Area, New Delhi 110016, India; 6 Public Health Foundation of India - Indian Institute of Public Health, Plot no # 1, Amar, Co-op Society, ANV Arcade, Madhapur, Kavuri Hills, Hyderabad 500033, India; 7 Department of Health & Family Welfare, Government of Andhra Pradesh, Koti, Sultan, Bazar, Hyderabad 500015, India; 8 State TB Office, Department of Health Services, Government of Andhra Pradesh, Koti, Sultan Bazar, Hyderabad 500015, India; 9 LEPRA India, Krishnapuri colony, West Marredpally, Hyderabad 500026, India; 10 Public Health Foundation of India - Indian Institute of Public Health, Plot no # 1, Amar Co-op Society, ANV Arcade, Madhapur, Kavuri Hills, Hyderabad 500033, India

**Keywords:** Tuberculosis, India, Death, Timing of death

## Abstract

**Background:**

India has 2.0 million estimated tuberculosis (TB) cases per annum with an estimated 280,000 TB-related deaths per year. Understanding when in the course of TB treatment patients die is important for determining the type of intervention to be offered and crucially when this intervention should be given. The objectives of the current study were to determine in a large cohort of TB patients in India:- i) treatment outcomes including the number who died while on treatment, ii) the month of death and iii) characteristics associated with "early" death, occurring in the initial 8 weeks of treatment.

**Methods:**

This was a retrospective study in 16 selected Designated Microscopy Centres (DMCs) in Hyderabad, Krishna and Adilabad districts of Andhra Pradesh, South India. A review was performed of treatment cards and medical records of all TB patients (adults and children) registered and placed on standardized anti-tuberculosis treatment from January 2005 to September 2009.

**Results:**

There were 8,240 TB patients (5183 males) of whom 492 (6%) were known to have died during treatment. Case-fatality was higher in those previously treated (12%) and lower in those with extra-pulmonary TB (2%). There was an even distribution of deaths during anti-tuberculosis treatment, with 28% of all patients dying in the first 8 weeks of treatment. Increasing age and new as compared to recurrent TB disease were significantly associated with "early death".

**Conclusion:**

In this large cohort of TB patients, deaths occurred with an even frequency throughout anti-TB treatment. Reasons may relate to i) the treatment of the disease itself, raising concerns about drug adherence, quality of anti-tuberculosis drugs or the presence of undetected drug resistance and ii) co-morbidities, such as HIV/AIDS and diabetes mellitus, which are known to influence mortality. More research in this area from prospective and retrospective studies is needed.

## Background

National TB Control Programmes (NTPs) routinely report treatment outcomes for patients with tuberculosis (TB). This is reflected in national reports and also annual reports from the World Health Organization (WHO) which provide data on treatment outcomes of TB patients from all countries in the world. Treatment outcomes include death, which is defined as death from any cause occurring during the course of anti-TB treatment.

There have been a few publications on the timing of death in patients while on treatment. In sub-Saharan Africa, studies have shown that the majority of deaths occur early during the first 1-2 months of anti-tuberculosis treatment [1-3]. In sub-Saharan Africa, where co-infection with HIV is high, this information has been important for NTPs to plan realistic strategies to reduce death rates during treatment. For example, giving antiretroviral therapy (ART) during the initial phase rather than the continuation phase of anti-tuberculosis treatment is more beneficial in reducing death rates in co-infected TB patients, and is based on a sound knowledge of when HIV-infected TB patients die [4,5]. Similar reports of early deaths have come from more industrialised countries such as Singapore [6], Russia [7] and Taiwan [8].

India, with a total population of 1.2 billion, has a well established national TB control programme, based firmly on the "DOTS" strategy, and treatment outcomes are reported regularly for patients with all types of TB [9]. India has an estimated 2.0 million incident TB cases each year, and an estimated TB-related mortality of 280,000 deaths per annum [9]. Understanding when in the course of TB treatment patients die is important for determining the type of intervention to be offered and crucially when this intervention should be given. Such information might be useful in further reducing case fatality rates among TB patients. There has been previous work carried out in India between 1999 and 2000 and in 2004 looking at timing of death as part of studies that assessed risk factors for death, failure and default, and between 50-65% of deaths were reported to occur within the initial phase of treatment [10,11]. However, each of these studies assessed less than 750 patients. As a result, there were small numbers of patients who died, and the findings may not be representative of the wider or more recent picture of timing of deaths during anti-tuberculosis treatment in India within the Revised National TB Control Programme (RNTCP).

The **aim **of this study was to document the timing of reported death in a large cohort of patients with tuberculosis who die during treatment. The **specific objectives **were to determine in a defined cohort of TB patients:- i) the treatment outcomes and the number who died while on treatment, ii) the month of death and iii) the characteristics of patients who were recorded as having died early in the initial 8 weeks of anti-tuberculosis treatment.

## Methods

### Study design and setting

This was a descriptive retrospective study based on record reviews and adhered to the methodological guidelines recommended in the STROBE document on observational studies [12]. The study was carried out in 16 selected Designated Microscopy Centres (DMCs) in Hyderabad, Krishna and Adilabad districts of Andhra Pradesh, South India. These centres were selected because activities are implemented by LEPRA India in partnership with 4 district TB control societies. TB treatment is initiated in India in accordance with the RNTCP DOTS strategy and, according to this strategy at the time; patients were placed on one of 3 categories for treatment [13]. The duration of treatment for patients in category 1, II or III varied from 6-8 months. Treatment outcomes were obtained for all patients registered during each quarter, 15 months after the start of treatment.

### Participants

All TB patients (adults and children) registered and placed on standardised anti-tuberculosis treatment in quarterly periods from January 2005 to September 2009 in the selected 16 DMCs situated in Hyderabad, Krishna and Adilabad in Andhra Pradesh, South India were included in the study.

### Source of data, variables and data collection instrument

Data were collected from the TB patient treatment card and follow-up records at the DMC, which in turn were cross checked with RNTCP Tuberculosis Unit TB registers. The following information was obtained:- TB registration number, age, sex, occupation, type of TB (Pulmonary TB- PTB and extra pulmonary TB - EPTB), category of treatment and HIV-serostatus. Treatment outcomes were recorded, including death during anti-TB treatment. In those who died, the timing of death was recorded as occurring at 4 week intervals from start to completion of treatment. A structured questionnaire was prepared and pilot tested in 10% of the patient records. This questionnaire was subsequently revised, and used to capture all data variables for the study.

### Analysis and statistics

Data were entered into an Excel file (MS Excel 2003), and were analysed using SPSS version 18 software. The chi-squared test was used to compare groups while the chi-square for trend was used to examine linear trends. Measures of risk were determined using odds ratios (OR) and 95% confidence intervals, with the level of significance set at *P *< 0.05.

### Ethics approval

This study proposal was approved by The Union Ethics Advisory Group, the Ethics committee of the Public Health Foundation of India and the LEPRA India - Institutional Ethical Committee. Permission was received from State TB Officer, Andhra Pradesh to carry out the study.

## Results

There were 8,240 TB patients (5183 men) whose mean age was 36 (SD 16) years. The treatment outcomes for all patients and also stratified by type and category of TB are shown in Table 1. Case fatality rates were 6% in patients with both new smear-positive and smear-negative pulmonary TB (PTB). Compared with patients who had new smear-positive PTB, case fatality rates were significantly lower at 1.6% in those with extra-pulmonary TB (EPTB) [RR 0.26, 95% CI 0.18-0.37, *P *< 0.001] and significantly higher at 12% in those with previously treated TB on a retreatment regimen [RR 1.93, 95% CI 1.58-2.36, *P *< 0.001].

**Table 1:** Treatment Outcomes in all TB patients and in relation to type and category of TB, Andhra Pradesh, India

All TB Patients	Registered on Treatment	Treatment success (%)	Death (%)	Default (%)	Transfer Out (%)	Failure (%)
NSP	3404	2996 (88%)	211 (6%)	153 (5%)	28 (< 1%)	16 (< 1%)

NSN	1663	1497 (90%)	103 (6%)	49 (3%)	11 (< 1%)	3 (< 1%)

EPTB	1945	1796 (92%)	31 (2%)	86 (4%)	31 (2%)	1 (< 1%)

Previously treated	1228	860 (70%)	147 (12%)	160 (13%)	32 (3%)	29 (2%)

**Total**	**8240**	**7149 (87%)**	**492 (6%)**	**448 (5%)**	**102 (1%)**	**49 (< 1%)**

*NSP *New Smear-Positive Pulmonary TB

*NSN *New Smear-Negative PTB

*EPTB *New Extra Pulmonary TB

Previously treated - previous TB which was treated with a standardised retreatment regimen

**Figure 1:**
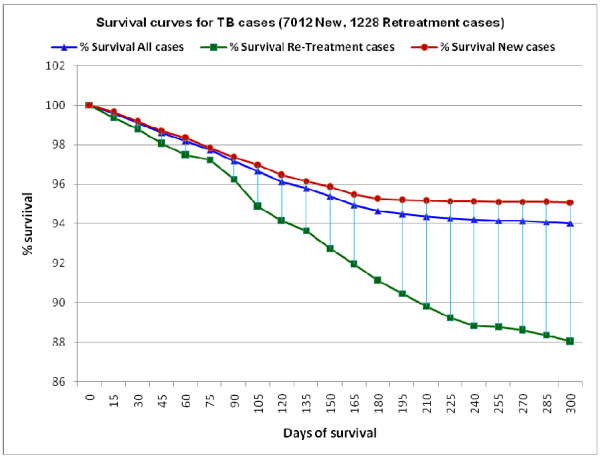
**Survival Curves for all TB patients and patients with new and retreatment TB**.

Timing of death in all TB patients during the course of anti-tuberculosis treatment is shown in Table 2, and the survival analysis curve with 3 line graphs stratified for all TB patients and patients with new and retreatment TB is shown in Figure 1. There was a fairly even distribution of deaths during the course of anti-tuberculosis treatment, with a lower proportion of patients dying in the four-week periods after 6 months (these patients were those on the longer retreatment regimens and those who were being treated for severe types of EPTB such as TB meningitis and TB spine). Patient characteristics associated with "early deaths" (i.e., dying in the first 8 weeks of treatment) are shown in Table 3. There was an increased odds of early death associated with age above 50 years, and in new patients and those treated with category 1 and 3 regimens there was an increased risk of early death compared with patients who had previously treated TB. Otherwise, gender, occupation and HIV status (including a comparison of HIV status known compared with HIV status not known) showed no significant association.

**Table 2:** Timing of death in patients who were recorded as having died during anti-tuberculosis treatment, Andhra Pradesh, India

Deaths reported in weeks from start of treatment	Deaths		Cumulative frequency	
	
	N	%	N	%
0-4 weeks	70	14	70	14

5-8 weeks	69	14	139	28

9-12 weeks	76	15	215	43

13-16 weeks	83	17	298	60

17-20 weeks	60	12	358	72

21-24 weeks	61	13	419	85

25-28 weeks	46	9	465	94

29 weeks and above	27	6	492	100.0

**Total**	**492**	**100**	**492**	**100**

**Table 3:** Characteristics of patients with "early" deaths reported during anti-tuberculosis treatment, Andhra Pradesh, India

Characteristics	Early deaths < 8 weeks		All deaths during treatment	Odds Ratio (95% Confidence Intervals)
**Gender**	**Number**	**%**	**Number**	

Male	102	28	363	OR 0.97 (0.6-1.6) *P *= 0.89

Female	37	29	129	Reference = 1

**Age**				

< 14 years	2	25	8	Chi-square test for trend = 5.668 *P *= 0.018
	
15-29 years	23	23	102	
	
30-49 years	55	26	216	
	
> 50+	59	36	166	

**Occupation**				

Skilled	11	18	61	Reference

Semi-skilled	22	28	79	OR 1.75 (0.7-4.3) P = 0.17

Manual	106	30	352	OR 1.96 (0.9-4.2) P = 0.05

**Type of TB**				

New smear-positive PTB	70	33	211	OR 2.0 (1.2-3.4) *P *< 0.01

New smear-negative PTB	28	27	103	OR 1.52 (0.8-2.9) *P *= 0.17

New EPTB	12	39	31	OR 2.57 (1.0-6.4) *P *= 0.02

Previously Treated TB	29	20	147	Reference = 1

**Treatment Category**				

Category 1 (New)	84	31	272	OR 1.82 (1.1-3.0) *P *= 0.01

Category 3 (New)	26	36	73	OR 2.2 (1.1-4.4) *P *= 0.01

Category 2 (Retreatment)	29	20	147	Reference = 1

**HIV status**				

HIV-positive	22	22	98	OR 0.82 (0.4-1.6) *P *= 0.53

HIV-negative	40	26	154	Reference = 1

HIV- status unknown	77	32	240	Not tested

**Total**	**139**	**28**	**492**	

## Discussion

This study in a large cohort of over 8,000 registered TB patients found a low case fatality at almost 6%. Patients with previously treated TB had higher death rates and those with EPTB had lower deaths rates than those with new pulmonary tuberculosis. The higher death rates in previously treated patients might be explained by more severe and drug resistant disease consequent upon failed first line therapy or initial and undiagnosed multi-drug resistant TB [13,14]. Although we did not document the types of EPTB during this study, the majority of patients in India with EPTB have lymph node disease [13], which tends to be associated with morbidity but not mortality.

There was a fairly even distribution of deaths during the course of anti-tuberculosis treatment. This is in marked contrast to the situation in sub-Saharan Africa where there is an excess of deaths in the first 1-2 months of treatment, thought to be due to late presentation and therefore severe tuberculosis disease as well as the effects of advanced HIV disease in those who are co-infected [3,4]. The two main characteristics associated with more frequent "early death" in this Indian study were age above 50 years and new disease compared with previously treated disease. Increasing age has been noted as a risk factor for death in other studies both within India [10,15] and outside of India [2], and new disease in contrast to recurrent disease was also associated with high early mortality during the first 4 weeks of treatment in Malawi [2]. Why this occurs is not known. Older people may be at higher risk of co-morbid disease which may result in a more serious illness at the time of presentation, diagnosis and treatment, and they may also develop chronic respiratory illnesses resembling and mistaken for smear-negative PTB due to chronic bronchitis and lung cancer [16]. Patients with recurrent disease may also be familiar with the symptoms and signs of TB, and therefore present earlier than those with new disease and as a result have less risk of early death. HIV-serostatus was not associated with "early deaths" in the small sample of patients who were HIV-tested, although many patients in the study were not HIV tested.

Why is there a difference in distribution of deaths in India compared with sub-Saharan Africa? First and most importantly, it will be necessary to repeat this study in other parts of the country and in large numbers of patients to ensure that the results in Andhra Pradesh are nationally representative. If indeed the results are confirmed, then there may be various explanations. India has a problem with initial defaulters [17] i.e., patients who are diagnosed with active TB but fail to get registered and placed on treatment. A high initial default rate will falsely lower early death rates in registered TB patients, obscuring the true picture of patients dying early during the registration and first few weeks of treatment of their disease. Second, HIV co-infection is lower in India than in Africa, with national rates of HIV-infection in TB patients currently at 5-10% [9]. Untreated, advanced HIV disease is therefore not an important factor in India, while in sub-Saharan Africa this has played a major role in case fatality and early deaths, and to some extent this has been mitigated by the introduction of cotrimoxazole preventive therapy and antiretroviral therapy.

If death rates are truly dispersed in an even manner during the course of anti-TB treatment in India then more work needs to be done in this area. Reasons may relate to the treatment of the disease itself, raising concerns about drug adherence, quality of anti-tuberculosis drugs or the presence of undetected drug resistance. They may also relate to co-morbidities which influence mortality. For example, diabetes mellitus has been calculated to account for 15%-20% of pulmonary TB in India [18], and there is growing evidence that diabetes is associated with an increased case fatality in TB patients [19-21]. The timing of death in diabetes patients who have TB is not known and requires active research, but one could speculate that diabetes exerts its negative effects throughout the course of anti-TB treatment as a result of drug-drug interactions, an increased association with anti-tuberculosis drug toxicity and immune suppressive effects of diabetes.

The strengths of this study are that there were a large number of patients enrolled in the cohort and treatment outcomes were tracked and recorded using standardised systems. However, there are a number of limitations. First, this was a record review and it is possible that mistakes were made in the recording of timing and date of death. Second, patients who are recorded as default or transfer out may also have died [15], and such misclassification may affect the results. Third, the records that formed the source of data did not contain valuable information such as results of any culture and drug sensitivity testing, which might have been important in explaining reasons for some of the deaths. Fourth, the 16 selected sites were all supported by LEPRA partnering with RNTCP, and as such may not be representative of other sites where this support was absent.

Whatever the shortcomings of this study, the results should serve to encourage others to repeat similar studies in other parts of the country and should also encourage programme staff to carefully record timing of death and timing of other adverse events such as default and transfer out. A number of potentially important measures might help to reduce death rates by ensuring that i) all patients diagnosed with smear-positive sputum are registered and start anti-TB treatment as soon as possible (thereby cutting down initial default rates) [22], ii) patients who are at risk of drug resistance, such as those previously treated, have culture and drug sensitivity testing so that treatment is appropriately tailored to levels of drug resistance, and iii) elderly patients are investigated for co-morbidities including diabetes mellitus which may increase the risk of death during treatment [23]. A better understanding of when and why the estimated 280,000 annual TB-related deaths occur is essential as India strives to improve programme performance and exceed the new 2015 global targets of treatment success rates of 90% in the years to come [24].

## Conclusions

In this large cohort of TB patients registered in Andhra Pradesh, South India, deaths occurred with an even frequency throughout anti-TB treatment. Reasons may relate to i) the treatment of the disease itself, raising concerns about drug adherence, quality of anti-tuberculosis drugs or the presence of undetected drug resistance and ii) co-morbidities, such as HIV/AIDS and diabetes mellitus, which are known to influence mortality. More research in this area from prospective and retrospective studies is needed.

## Competing interests

The authors declare that they have no competing interests.

## Authors' contributions

SJ conceived the study and participated in its design, data collection, analysis, interpretation of the data and draft the manuscript. ADH, RZ, SS and ST participated in the study design and coordination and helped to draft the manuscript by providing critical important intellectual inputs for finalisation. KCG, SRMS, RA and NKK have been involved in designing and drafting the manuscript by providing critical inputs. RRPV and SP participated in the design of the study and performed the statistical analysis. All authors have read and approved the final manuscript.

## Pre-publication history

The pre-publication history for this paper can be accessed here:

http://www.biomedcentral.com/1471-2458/11/921/prepub
